# Safety and feasibility of initiating peritoneal dialysis within 24 h after percutaneous versus surgical catheter placement

**DOI:** 10.1093/ckj/sfag080

**Published:** 2026-03-18

**Authors:** Brenda Guadalupe Cortez-Flores, Cesar R Barrientos, Pablo Galindo, Rosario G Hernández, Juan Reyna-Blanco, Rubén Garrido Roldán, Héctor Benjamín García Aguilar, J Sergio Barrón Morales, J Emilio Sanchez-Alvarez, Bernardo Moguel-González

**Affiliations:** Department of Nephrology, Instituto Nacional de Cardiología Ignacio Chavez, Mexico City, Mexico; Department of Nephrology and Mineral Metabolism, Instituto Nacional de Ciencias Médicas y Nutrición Salvador Zubirán, Mexico City, Mexico; Department of Nephrology, Hospital General de México Dr. Eduardo Liceaga, Mexico City, Mexico; Department of Nephrology and Mineral Metabolism, Instituto Nacional de Ciencias Médicas y Nutrición Salvador Zubirán, Mexico City, Mexico; Department of Nephrology, Instituto de Seguridad Social del Estado de México y Municipios, Ecatepec, Mexico; Department of Nephrology, Hospital General de México Dr. Eduardo Liceaga, Mexico City, Mexico; Department of Nephrology, Instituto Nacional de Cardiología Ignacio Chavez, Mexico City, Mexico; Department of Nephrology, Instituto Nacional de Cardiología Ignacio Chavez, Mexico City, Mexico; Department of Nephrology, Instituto de Seguridad Social del Estado de México y Municipios, Ecatepec, Mexico; Hospital Universitario Central Asturias, Oviedo, Spain; Department of Nephrology, Instituto Nacional de Cardiología Ignacio Chavez, Mexico City, Mexico

**Keywords:** break-in period, percutaneous placement, peritoneal dialysis, urgent-start PD

## Abstract

**Background:**

End-stage kidney disease (ESKD) is a rising global health challenge. Despite superior quality of life and cost-effectiveness, only 11% of patients initiate renal replacement therapy with peritoneal dialysis (PD), primarily due to the traditional extended break-in period. Recent advancements in percutaneous catheter placement have facilitated urgent-start and early-start PD protocols. This study compared the break-in period [immediate-start PD (<24 h)] and catheter placement–related complications between percutaneous and surgical techniques.

**Methods:**

This retrospective multicenter study analyzed 429 patients who underwent PD catheter insertion at three Mexican nephrology centers; 416 patients had confirmed ESKD. Techniques included mini-laparotomy and percutaneous methods (stylet or Seldinger). Data collection encompassed baseline characteristics, break-in periods and 30-day complications, classified as minor (mechanical and infectious) or major (bleeding, visceral perforation and death).

**Results:**

The majority of patients initiated PD within 24 h of catheter placement, and 49% within 6 h, 88% of patients using the Stylet technique and 58% of those using the Seldinger technique, compared with 3.8% using mini-laparotomy (*P* < .001). Percutaneous techniques represented 97% of the break-in periods within 6 h, whereas mini-laparotomy accounted for 91% of the break-in periods between 25 and 72 h. Complications affected 30% of the patients overall, with mechanical issues (23%) predominating, including outflow failure (19%) and catheter migration (9.1%), with no differences observed between techniques (*P* = .9, *P* = .8). The rate of repositioning was greater with the use of percutaneous methods (13%–14% vs 4%, *P* = .008). Infections were more common with mini-laparotomy (14% vs 7.4%–8.3%, *P* = .12), with exit-site infections (8.1% vs 1.4%–2.2%, *P* = .006) and tunnelitis (6.0% vs 0.7%–2.8%, *P* = .041) exhibiting significance. Mini-laparotomy increased the risk of infection (odds ratio 2.13, 95% confidence interval 1.02–4.44; *P* = .043).

**Conclusion:**

Immediate-start PD, defined as initiation within 24 h of catheter placement, is safe and feasible. These findings support the expansion of nephrologist training in percutaneous placement.

KEY LEARNING POINTS **What was known:**

- Around 50% of patients with end-stage chronic kidney disease begin renal replacement therapy without prior planning.
- Peritoneal dialysis (PD) typically has a 14-day break-in period after catheter placement, usually by open surgical and blind percutaneous methods.
- In low- and middle-income countries late referrals and unplanned dialysis starts are frequent.

**This study adds:**

- Percutaneous placement consistently enabled an earlier break-in time, while mini-laparotomy tended to require longer intervals.
- Mechanical complications and major events were uncommon across different placement techniques.
- Adjusted analyses suggested higher infectious risk with surgical placement.

**Potential impact:**

- Findings can be translated to everyday services, supporting nephrologist-led percutaneous training and broader access to urgent/immediate-start PD.
- Our data can inform access pathways, training curricula and ongoing discussions about break-in periods.

## INTRODUCTION

End-stage kidney disease (ESKD) has become one of the fastest-growing diseases over the past 20 years [1]; Mexico is among the countries with the highest incidence of ESKD in Latin America [2].

Although peritoneal dialysis (PD) is associated with multiple advantages for patients, such as a better lifestyle and improved quality of life, prolonged preservation of residual kidney function, and low-cost accessibility, only 11% of patients initiate renal replacement therapy (RRT) with PD [3, 4].

Approximately 40%–60% of patients with ESKD begin RRT without prior planning [4, 5]. PD is the least preferred modality, and one of the most important factors is that the traditional break-in period is at least 14 days [6]. However, PD has recently become an attractive alternative for unplanned RRT initiation. This is due mainly to the growth of interventional nephrology training programs and the redefinition of novel percutaneous placement techniques. Blake introduced the term “early start PD” for patients who start treatment between 3 and 14 days after catheter placement and “urgent-start PD” for those who start treatment during the first 72 h [7].

One of the most relevant factors in the short and medium term for successful PD treatment is the catheter insertion technique [8]. Open surgical and blind percutaneous techniques are the most frequently used [9, 10].

To date, no studies have compared the success rate and complications of the catheter insertion technique in the setting of urgent-start PD.

In this study, we evaluated the break-in period and catheter placement-related complications in patients undergoing urgent-start PD with shorter break-in periods (<24 h) and compared the surgical technique (mini-laparotomy), Seldinger and stylet technique.

## MATERIALS AND METHODS

### Study design and population

This was a retrospective multicentric study that included all PD catheter insertions performed from April 2019 to April 2024 at three different interventional nephrology centers in Mexico: Instituto Nacional de Cardiología Dr Ignacio Chávez, Centro Médico ISSEMYM Ecatepec and Hospital General de México Dr Eduardo Liceaga. Patients were followed for 30 days after the procedure or insertion. As a part of the PD programs, these centers prospectively recorded comorbidities; PD initiation criteria; laboratory tests at the time of catheter placement; break-in periods; and mechanical, infectious and major complications. The data for this study were obtained from PD program records.

### Eligibility

Patients were included in the analysis if they were 18 years of age or older and had a PD catheter placed for any indication via either a percutaneous or surgical technique. Patients were excluded if they were under 18 years of age, if their data records were incomplete or if follow-up was lost within 30 days of catheter placement.

### Catheter placement

The indication for PD catheter placement and the type of placement technique was established by the nephrologist in charge, and the decision was influenced mainly by the severity of dialysis indications, the indication for urgent-start PD, the history of abdominal surgeries, the body mass index and the previous failure of a percutaneous technique.

#### Mini-laparotomy

The surgical technique was performed by surgeons in an operating room via an open laparotomy approach with direct visualization of the peritoneal cavity and patients undergoing regional anesthesia or mild sedation. A 57-cm curled catheter (Argyle Curl Cath, 2-cuff, Vantive US Healthcare) was inserted under direct vision and positioned in the pelvic cavity. All patients underwent bowel preparation, were required to fast before the procedure, and received prophylactic antibiotics either before or within 2 h after the procedure.

#### Percutaneous techniques

All percutaneous catheter placements were performed by trained interventional nephrologists, who used only local anesthesia either in the procedure room or at the patient’s bedside. These procedures did not require any special preparation or fasting, particularly in cases where there was an indication for urgent start. Prior to placement, all patients were evaluated by ultrasound to confirm complete bladder emptying, identify the epigastric artery territory, identify adequate peritoneal sliding and measure the distance from the skin to the peritoneal cavity.

The insertion site was identified by aligning the catheter coil with the pubic symphysis, as previously described [11]. Local anesthesia was given via the use of 2% lidocaine and ultrasound guidance for infiltration of the skin, subcutaneous tissue, muscular layers and parietal peritoneum. A 1-cm skin incision was then made at the planned insertion site, and access to the peritoneal cavity was achieved via one of two percutaneous techniques: the stylet technique or the Seldinger technique. All patients received antibiotic prophylaxis as previously described for the surgical technique.

For the percutaneous stylet technique, blunt dissection of the subcutaneous tissue was performed via clamps or forceps up to the aponeurosis. Once the fascia was identified, access to the peritoneal space was achieved via blunt-tipped forceps to create a 2–5 mm space. A 57-cm curled catheter (Argyle Curl Cath, 2-cuff, Vantive US Healthcare) was then inserted and advanced into the pelvis via a 61 cm metallic stylet (Vantive US Healthcare), with the deeper cuff positioned in the preperitoneal space.

For the Seldinger technique, preassembled kits were used (Argyle Curl Cath Kit, 2-cuff, 16 Fr, or Merit Medical Flex-Neck Classic, 2-cuff, adult standard, 18 Fr; Vantive US Healthcare). Peritoneal cavity access was achieved via real-time ultrasound-guided puncture. Confirmation of cavity entrance was performed with saline contrast agent and color Doppler distribution in the peritoneal space. A guidewire was then introduced under ultrasound guidance with further dilatation over the guidewire with a 16 Fr or 18 Fr dilator, depending on the kit. A peel-away sheath was placed into the peritoneal space, and the guidewire was removed. Finally, a 57-cm curled catheter was inserted and advanced into the pelvis, with the deeper cuff positioned above the fascia.

#### Functionality test and catheter confirmation

After placement and before catheter tunnel creation, 2 L of dialysis fluid was introduced into the peritoneal cavity and drained to confirm functionality. An abdominal X-ray was obtained to verify the location of the catheter.

#### Subcutaneous tunneling and wall closure

The subcutaneous tunnel creation technique was identical across all three placement methods, utilizing a tunneler and planned according to the incision site, catheter exit location and position of the second subcutaneous cuff. In the mini-laparotomy approach, both fascial and skin closure were performed with sutures, whereas the percutaneous techniques required only skin suturing.

#### Dialysis initiation

For mini-laparotomy, dialysis was typically started after 24–48 h but could be initiated earlier in emergencies. The percutaneous technique allowed for immediate initiation (<24 h) on the basis of physician discretion. The dialysis volume was calculated as 30 mL/kg per exchange or 1500 mL/m^2^ of body surface area per exchange and then rounded to the nearest whole number. All initial dialysis prescriptions were initiated using the full target volume. Mobilization was implemented according to their clinical condition; all patients received the standard care for PD.

### Follow-up

#### Follow-up time baseline characteristics and break-in period

A 30-day follow-up period was used to assess baseline characteristics, comorbidities, surgical history, biochemical parameters, volume status, break-in period (defined as the time from catheter insertion to full dialysis prescription) and complications.

### Complications

#### Mechanical complications

Outflow failure was defined as either a lower volume of fluid recovered during drainage than the volume infused and/or a drainage time to achieve complete outflow exceeding 30 min. Fluid leakage was defined as any loss of dialysate from the peritoneal cavity through the incision site or subcutaneous tunnel. Catheter migration was established if dysfunction of the catheter was accompanied by abdominal X-rays showing the curled tip positioned outside the pelvic cavity after baseline imaging confirmed correct initial placement. Repositioning was defined as the need to repeat the procedure after initial successful placement, requiring reinstallation of the catheter.

#### Infectious complications

Infectious complications were defined according to the criteria established by the International Society for Peritoneal Dialysis (ISPD) [12]. The diagnosis of peritonitis required the presence of two out of the following three criteria: (i) clinical features consistent with peritonitis, such as abdominal pain and/or cloudy PD effluent; (ii) a peritoneal effluent white blood cell count >100 cells/μL after a dwell time of at least 2 h, with at least 50% polymorphonuclear neutrophils; and (iii) a positive culture from the PD effluent. Exit-site infection was defined as the presence of purulent drainage with or without erythema at the catheter exit site. Tunnel infection (tunnelitis) was defined as the presence of tenderness, erythema or swelling along the subcutaneous tunnel of the catheter, associated with sonographic features compatible with the diagnosis, such as fluid collection along the tunnel or augmented vascularity, with or without purulent discharge at the exit site.

#### Major complications

Bladder or bowel perforation, major bleeding and death related to catheter placement were classified as major complications. Major bleeding was defined as any hemorrhage associated with catheter placement causing hypotension, hypovolemic shock or vasopressor initiation, and requiring blood product transfusion or surgical intervention for its resolution.

### Statistical analysis

Continuous variables were evaluated via the Shapiro‒Wilk test. Descriptive statistics are reported as frequencies (percentages), medians (interquartile ranges) or means ± standard deviations, as appropriate. Between-group comparisons were performed via chi-square, Fisher’s exact or Kruskal‒Wallis tests, depending on the data type. Also, univariate and multivariate logistic regression analyses were performed to determine risk factors for infection, including age, sex and type of catheter placement technique. Analyses were conducted via R version 4.3.2.

### Ethical considerations

This study was conducted in accordance with the Declaration of Helsinki and approved by the ethics committees of the following institutions: Centro Medico ISSEMYM Ecatepec (approval number: PICME (2023/10)), Instituto Nacional de Cardiologia “Dr Ignacio Chavez” (approval number: CI-078-2025) and Hospital General de Mexico “Dr Eduardo Liceaga” (approval number: DECS/JPO-CT-3326-2025).

## RESULTS

**Figure 1:**
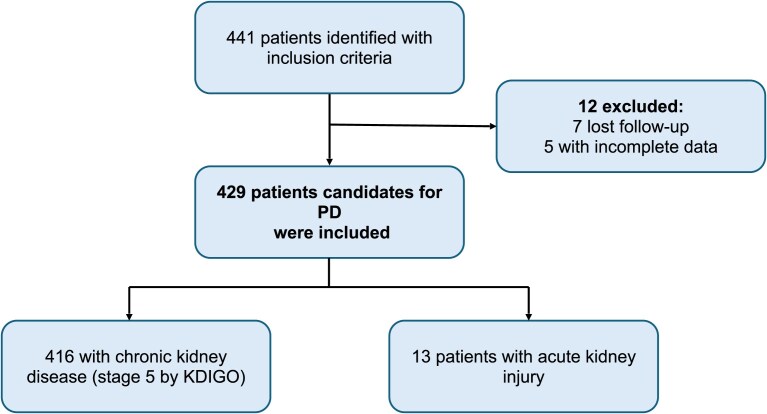
Flow diagram of patient selection for the study. Of 441 patients initially identified, 12 were excluded (7 lost to follow-up, 5 with incomplete data). A total of 429 patients candidates for PD were included: 416 with chronic kidney disease stage 5 and 13 with acute kidney injury.

A total of 441 patients meeting the inclusion criteria were identified. Twelve patients were excluded: seven due to loss to follow-up (transfer to another unit) and five due to incomplete clinical data. A total of 429 patients were ultimately included in the study, 416 patients with ESKD (chronic kidney disease stage 5, as defined by KDIGO) and 13 patients with acute kidney injury who met the criteria for the initiation of RRT and were considered candidates for PD catheter placement (Fig. 1).

### Baseline characteristics

The population was predominantly men (60%), with significant differences in sex distribution between the techniques used (*P* = .015). The median age was 56 years (interquartile range 43–64), with no significant differences between the groups (*P* = .090). The most common comorbidity was hypertension (66%), followed by type 2 diabetes (63%). Fifty-one percent had a history of abdominal surgery, with a higher prevalence in the mini-laparotomy group (77%) than in the Seldinger (39%) and stylet (36%) groups (*P* < .001).

At the time of placement, 76% of patients had blood urea nitrogen >100 mg/dL, 36% had hyperkalemia, 47% had metabolic acidosis and 44% had volume overload. Regarding dialysis initiation, 41% were planned, 30% were unplanned (clinical or biochemical criteria for urgent dialysis) and 28% initiated hemodialysis upon admission by medical decision (Table 1).

**Table 1:** Baseline characteristics.

		Placement technique			
Characteristic	Overall (*N* = 429^^a^^)	Mini-lap (*N* = 149^^a^^)	Seldinger (*N* = 135^^a^^)	Stylet (*N* = 145^^a^^)	*P*-value^^b^^
Sex (*n*, %)					.015
Female	173 (40)	74 (50)	49 (36)	50 (34)	
Male	256 (60)	75 (50)	86 (64)	95 (66)	
Age (years)	56 (43, 64)	58 (44, 66)	54 (42, 62)	53 (43, 63)	.090
Type 2 diabetes (*n*, %)	239 (63)	76 (60)	78 (64)	85 (65)	.7
Hypertension (*n*, %)	73 (66)	41 (68)	32 (65)	0 (0)	.4
Prior abdominal surgery (*n*, %)	219 (51)	115 (77)	52 (39)	52 (36)	<.001
Biochemical characteristics at time of placement					
Hyperkalemia (>5.5 mEq/L) (*n*, %)	146 (36)	49 (38)	47 (36)	50 (34)	.9
Urea nitrogen >100 mg/dL (*n*, %)	326 (80)	104 (80)	87 (66)	135 (93)	<.001
Acidosis (*n*, %)	188 (47)	58 (46)	58 (44)	72 (50)	.7
Bicarbonate levels (mEq/L)	14 (0, 20)	13 (0, 18)	16 (4, 21)	13 (0, 19)	.062
Fluid overload (*n*, %)	178 (44)	37 (28)	75 (57)	66 (46)	<.001
Starting criteria for PD (*n*, %)					
Planned start	178 (41)	66 (44 )	38 (28 )	74 (51)	<.001
Unplanned start	130 (30 )	20 (13)	51 (38 )	59 (41 )	<.001
Started with hemodialysis	121 (28)	63 (42)	46 (34)	12 (8.3)	<.001
Complications (*n*, %)					
Presence of any complication	130 (30)	51 (34)	34 (25)	45 (31)	.2
Major complications	9 (2.1)	4 (2.7)	1 (0.7)	4 (2.8)	.4
Infectious complications	43 (10)	21 (14)	10 (7.4)	12 (8.3)	.12
Mechanical complications	97 (23)	33 (22)	29 (21)	35 (24)	.9

a *n* (%); median (Q1, Q3).

b Pearson’s Chi-squared test; Kruskal–Wallis rank sum test; Fisher’s exact test.

#### Break-in period and placement technique

Overall, 73% of patients-initiated PD within 24 h of catheter placement, with nearly half (49%) of patients-initiating catheter use within the first 6 h after placement, with this proportion being significantly greater in the groups treated with percutaneous techniques: stylet (89%) and Seldinger (57%). In contrast, only 3.4% of patients treated with the mini-laparotomy technique experienced an early break-in period (*P* < .001).

**Figure 2:**
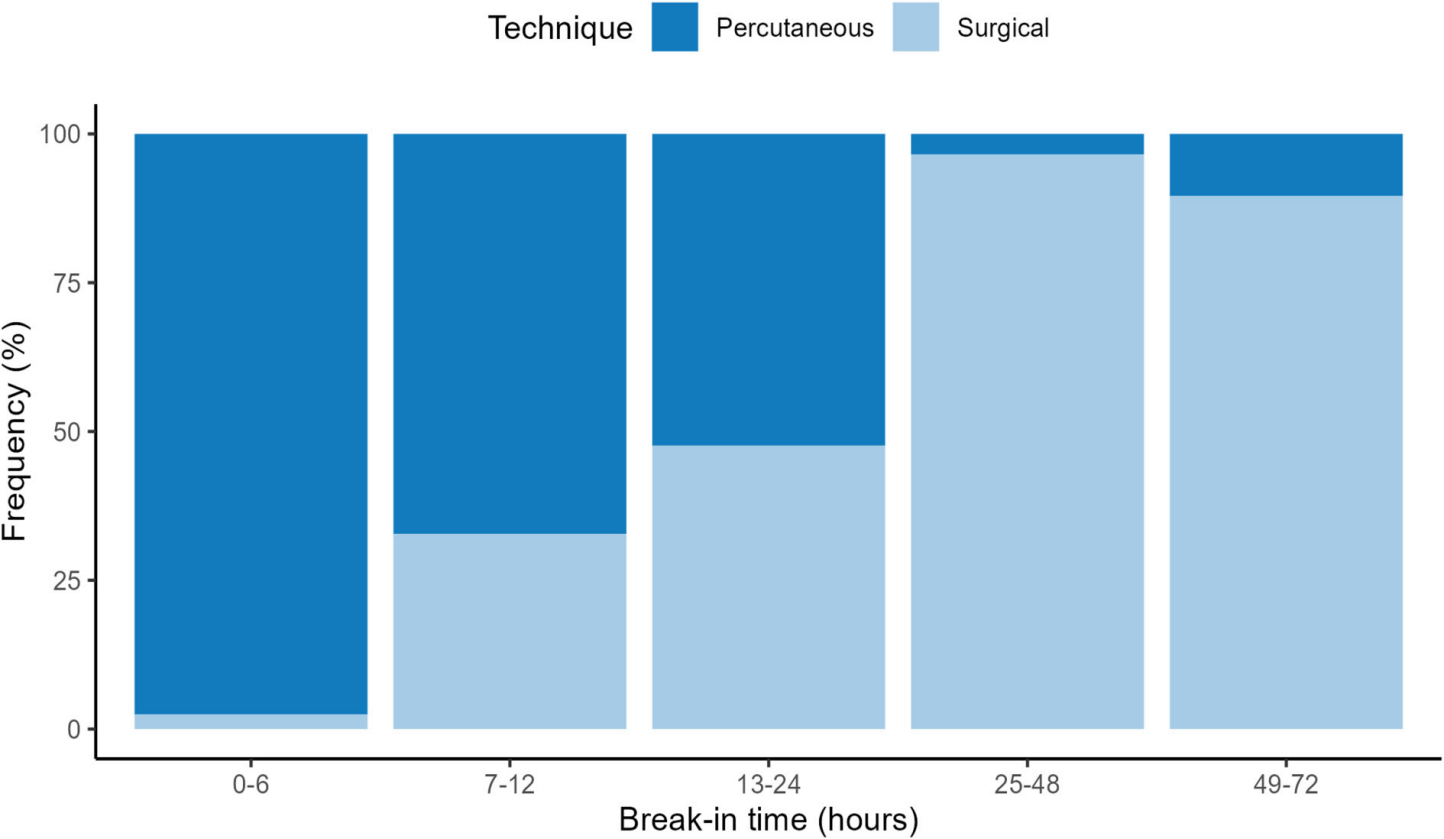
Break-in time according to placement technique: surgical (mini-lap) vs percutaneous (Seldinger and stylet). Distribution of break-in time intervals (0–6 h, 7–12 h, 13–24 h, 25–48 h and 49–72 h) by placement technique. The surgical group (mini-laparotomy) showed a predominance in delayed use (25–72 h), while the percutaneous techniques (Seldinger and stylet) were associated with early catheter use, particularly within the first 6 h post-insertion (*P* = .001).

Ninety-seven percent of break-in periods within the first 6 h occurred with the percutaneous technique. In contrast, the mini-laparotomy technique was associated with a longer break-in period, accounting for 91% of the break-in periods occurring between 25 and 72 h, with statistically significant differences between groups (*P* < .001) (Fig. 2 and Table 2).

**Table 2:** Break-in period.

		Placement technique			
Characteristic	Overall (*N* = 429^^a^^)	Mini-lap (*N* = 149^^a^^)	Seldinger (*N* = 135^^a^^)	Stylet (*N* = 145^^a^^)	*P*-value^^b^^
0–6 h	211 (49)	5 (3.4)	77 (57)	129 (89)	.001
7–12 h	64 (15)	20 (13)	39 (29)	5 (3.4)	.001
13–24 h	42 (9.8)	20 (13)	12 (8.9)	10 (6.9)	.001
25–48 h	39 (9.1)	38 (26)	1 (0.7)	0 (0)	.001
49–72 h	73 (17)	66 (44)	6 (4.4)	1 (0.7)	.001

a *n* (%).

b Pearson’s Chi-squared test; Kruskal–Wallis rank sum test; Fisher’s exact test.

One hundred of patients with acute kidney injury started therapy within the first 6 h after catheter placement (*P* = .014), all of whom were placed with percutaneous technique—77% with Seldinger and 23% with stylet.

#### Complications related to catheter placement

Thirty percent of patients experienced at least one complication during the procedure or in the first 30 days after the procedure. The most frequent complications were mechanical complications (23%), followed by infections (10%) and major complications (2.1%) (Table 1). There were no differences between patients with acute kidney injury or chronic kidney disease.

### Mechanical complications

Outflow failure was the most frequent mechanical complication reported in 19% of cases, with no significant differences between techniques (*P* = .9) (Table 3). Catheter migration occurred in 9.1% of patients, with no differences between the groups (*P* = .8). However, the need for repositioning was significantly greater in the Seldinger (13%) and stylet (14%) groups than in the mini-laparotomy (4%) group (*P* = .008). Fluid leakage was infrequent (2.6%), with no significant differences between techniques (*P* = .6).

**Table 3:** Mechanical complications.

		Placement technique			
Characteristic	Overall (*N* = 429^^a^^)	Mini-lap (*N* = 149^^a^^)	Seldinger (*N* = 135^^a^^)	Stylet (*N* = 145^^a^^)	*P*-value^^b^^
Outflow failure	83 (19)	27 (18)	27 (20)	29 (20)	.9
Migration	39 (9.1)	12 (8.1)	12 (8.9)	15 (10)	.8
Repositioning	44 (10)	6 (4.0)	18 (13)	20 (14)	.008
Fluid leakage	11 (2.6)	4 (2.7)	2 (1.5)	5 (3.4)	.6

a *n* (%).

b Pearson’s Chi-squared test; Kruskal–Wallis rank sum test; Fisher’s exact test.

### Infectious complications

Infectious complications occurred in 10% of the patients (Table 4). Although no statistically significant differences were observed overall (*P* = .12), the mini-laparotomy group had the highest rate (14%), followed by the stylet (8.3%) and Seldinger (7.4%) groups. Exit-site infection was more common in the mini-laparotomy group (8.1%) than in the Seldinger (2.2%) and stylet (1.4%) groups, with significant differences (*P* = .006). The rate of tunnelitis was also greater in the mini-laparotomy group (6.0%) than in the Seldinger (0.7%) and stylet (2.8%) groups (*P* = .041). The incidence of peritonitis was 4.2%, with no difference between techniques (*P* = .7%).

**Table 4:** Infectious complications.

Characteristic		Placement technique			
	**Overall (*N* = 429** ^**^a^**^)	**Mini-lap (*N* = 149** ^**^a^**^)	**Seldinger (*N* = 135** ^**^a^**^)	**Stylet (*N* = 145** ^**^a^**^)	***P*-value** ^**^b^**^
Infectious complications	43 (10)	21 (14)	10 (7.4)	12 (8.3)	.12
Exit-site infection	17 (4.0)	12 (8.1)	3 (2.2)	2 (1.4)	.006
Tunnelitis	14 (3.3)	9 (6.0)	1 (0.7)	4 (2.8)	.041
Peritonitis	18 (4.2)	5 (3.4)	7 (5.2)	6 (4.2)	.7

a *n* (%).

b Pearson’s Chi-squared test; Kruskal–Wallis rank sum test; Fisher’s exact test.

In the univariable analysis, mini-laparotomy placement was associated with a slightly increased but statistically significant risk of infectious complications [odds ratio (OR) 1.09; 95% confidence interval (CI) 1.02–1.16; *P* = .006]. When adjusting for other clinical variables in the multivariable model, the association became more pronounced (OR 2.13; 95% CI 1.02–4.44; *P* = .043), indicating that mini-laparotomy may independently contribute to a greater risk of infections.

### Major complications

The overall major complication rate was 2.1% (Table 1). Significant bleeding occurred in four patients (0.9%) and bladder injuries were observed in 0.7% of patients. Intestinal perforation was reported only in the stylet group (1.4%). Additionally, one case of intraprocedural mortality (0.2%) was recorded in a patient with a mechanical valve, active endocarditis and systemic anticoagulation, also in the stylet group. There was no statistically significant difference between the techniques regarding these types of complications (Table 5).

**Table 5:** Major complications.

		Placement technique			
Characteristic	Overall (*N* = 429^^a^^)	Mini-lap (*N* = 149^^a^^)	Seldinger (*N* = 135^^a^^)	Stylet (*N* = 145^^a^^)	*P*-value^^b^^
Major bleeding	4 (0.9)	3 (2.0)	0 (0)	1 (0.7)	.3
Bladder injury	3 (0.7)	1 (0.7)	1 (0.7)	1 (0.7)	>.9
Bowel perforation	2 (0.5)	0 (0)	0 (0)	2 (1.4)	.2
Death	1 (0.2)	0 (0)	0 (0)	1 (0.7)	.7

a *n* (%).

b Pearson’s Chi-squared test; Kruskal–Wallis rank sum test; Fisher’s exact test.

## DISCUSSION

In our study with more than 400 patients included we showed that immediate-start PD with percutaneous techniques is safe and feasible, with break-in periods as short as 6 h and low complication rates. One of the most critical factors influencing the success and viability of urgent-start PD is the resting period between catheter insertion and the initiation of therapy—commonly referred to as the “break-in period” [6]. Most published cohorts reported a break-in period of at least 24 h. For example, in a study by Xu *et al*., only 17% of patients initiated PD within the first 24 h [13]. Similarly, a Mexican cohort reported that 77% of patients started dialysis after 24 h, whereas only 13% initiated therapy earlier [14].

In contrast, our study demonstrated a considerably shorter break-in period: nearly 80% of patients began dialysis within 24 h, and >60% did so within 12 h. This accelerated initiation was associated primarily with the use of percutaneous catheter placement performed by nephrologists. Specifically, 86% of patients with percutaneous placement-initiated dialysis within 12 h, whereas only 16% of those with surgical placement did.

Although urgent-start PD protocols often recommend beginning therapy with low dialysate volumes, our cohort was treated with standard volumes—averaging 2 L per exchange [15, 16]. Despite this, abdominal wall complications were uncommon and mild, with no cases of hydrothorax or PD-related hernias. Previous studies have reported dialysate leakage rates ranging from 6% to 8%, with initiation typically occurring approximately 48 h post-placement [13, 15]. In contrast, our leakage rate (2.6%) was the lowest reported to date in the context of urgent-start PD, even though dialysis in most patients was initiated within 24 h and using high infusion volumes. We hypothesize that this outcome is related to the use of percutaneous techniques, in which a 5 mm access perforation was created without forcing the deep cuff into the fascia, resulting in a tract closely matched to the catheter diameter and thereby reducing the likelihood of leakage. These findings suggest that immediate use of the peritoneal cavity is both safe and feasible when percutaneous techniques are employed.

No significant differences were found in the incidence of mechanical complications between the techniques, despite the larger incisions typically required in surgical approaches. Furthermore, the need for a follow-up intervention (such as transition to hemodialysis, catheter replacement or repositioning) was notably lower (17%) than that reported in previous urgent-start PD studies, such as 40% reported by Alves *et al*. [15]. Interestingly, this rate was comparable to that reported in elective, ambulatory patients without prior abdominal surgery (17% vs 15.5%) [17]. These findings reinforce the notion that, even in patients with urgent dialysis indications, mechanical complications are uncommon and often manageable through conservative means (e.g. heparinization, flushing protocols or laxatives).

A prior study suggested that catheter placement by nephrologists might be associated with a greater risk of secondary interventions [17]. Our results did not replicate this finding, a discrepancy that may be attributed to the technical approach used rather than the provider’s specialty. In our cohort, surgeons exclusively employed the open surgical technique (34% of cases), whereas nephrologists exclusively used percutaneous techniques (66%). Conversely, in the cited study surgeons predominantly used laparoscopic and percutaneous methods.

A substantial proportion of our cohort had a history of previous abdominal surgery. As reported in the literature, we observed an incremental increase in mechanical complications correlated with the number of prior surgeries—primarily in the form of drainage dysfunction and catheter migration. Nevertheless, most of these complications were effectively managed conservatively. The need for a second procedure was significantly greater in patients who underwent two or more prior abdominal surgeries.

Infectious complications are a key concern in urgent-start PD [18, 19]. In our study, percutaneous insertion was associated with fewer exit-site and tunnel infections, consistent with prior evidence from earlier reports favoring percutaneous over surgical techniques despite methodological limitations [20–22]. Meta-analyses have also shown higher risks of exit-site, tunnel infection and peritonitis with open surgical placement [23, 24]. Yet, studies focused specifically on urgent unplanned settings remain scarce and have reported no clear differences between techniques [25, 26]. Our findings are the first to demonstrate reduced catheter-related infections with percutaneous insertion in the urgent-start context. Considering that the percutaneous technique is less traumatic and requires a smaller incision—features associated with reduced postoperative leakage—these findings are consistent with its inherent procedural advantages.

Based on our findings and procedural protocols, we propose the term “immediate-start PD” to refer to the initiation of PD within 24 h in patients without prior preparation and with urgent indications for RRT.

Importantly, all the participating centers were academic institutions with extensive experience in percutaneous catheter placement and PD programs managed by nephrologists. Therefore, these results may be most applicable to centers with similar expertise and infrastructure.

### Limitations

Several limitations to this study should be acknowledged. First, although patient weight was available for calculating initial dialysis volumes, height was not systematically recorded across all three centers, which precluded the calculation of body mass index as a variable in our analysis. Second, the retrospective design of this study inherently limits the ability to establish definitive causal or temporal relationships between the placement techniques and the observed outcomes. As with all retrospective studies, there is a potential for selection bias and the omission of clinical variables not routinely captured in medical records. Third, the expertise of the operators may have influenced the results. While all three participating centers are high-volume academic institutions, the interventional nephrologists at the Instituto Nacional de Cardiología possess more years of experience, as it is the founding site for these procedures. Additionally, because these are teaching hospitals, many procedures were performed by fellows under the supervision of a senior tutor, a factor that could impact procedural duration or complication rates. Finally, the follow-up period was limited to 30 days post-catheter insertion. While this timeframe is critical for assessing immediate-start PD complications, the long-term sustainability of these results and the incidence of late-onset complications remain unknown. Future prospective, longitudinal studies are required to confirm these findings and assess long-term outcomes beyond the initial break-in period.

## CONCLUSIONS

Immediate-start PD is feasible. Percutaneous technique is associated with lower complication rate compared with mini-laparotomy and appears safe, with complications rates comparable to elective PD. The findings suggest broader use of percutaneous PD catheter placement rather than immediate-start PD protocols.

## Data Availability

The data underlying this article will be shared on reasonable request to the corresponding author.
